# The Role of Political Belief in COVID-19 Vaccine Resistance, Virus Transmission, and Closure Policy Response

**DOI:** 10.3390/vaccines11061046

**Published:** 2023-05-31

**Authors:** Danny Ben-Shahar, Stuart Gabriel, Roni Golan

**Affiliations:** 1 Coller School of Management, Tel Aviv University, Tel Aviv 6139001, Israel; bdanny@tauex.tau.ac.il (D.B.-S.); roni.golan@niceactmize.com (R.G.); 2 Anderson School of Management, University of California, Los Angeles, CA 90095-1481, USA; 3 NICE Actimize, Ra’anana 4366241, Israel

**Keywords:** political belief, health risk, vaccine resistance, policy response

## Abstract

We employ unique panel data on the universe of COVID-19 vaccination and infection cases in Israel to examine the role of political belief in COVID-19 vaccine uptake, virus transmission, and closure policy response. The paper identifies political beliefs based on statistical area votes in national elections held in Israel on the eve of the COVID-19 outbreak in March 2020. Unlike the U.S. and elsewhere, pandemic policy intervention in Israel was broadly supported by politicians across the belief spectrum. As such, household response to virus risk was not biased by contemporaneous partisan disagreement and debate among political leaders. Findings show, all things equal, that in the wake of emergent and localized virus risk, voters in politically right-of-center and religious areas displayed substantially higher odds of both vaccine resistance and virus transmission as compared to their left-center counterparts. Moreover, political belief is highly salient to aggregate pandemic outcomes. Model simulation shows that had all areas responded to virus risk with the more risk-averse behaviors of left-of-center areas, the number of vaccinations nationwide would have increased by 15 percent. That same scenario results in a full 30 percent reduction in total infection cases. Results also show that coercive policy measures such as economic closure were more effective in reducing virus transmission among less risk-averse right-wing and religious areas. Findings provide new evidence of the role of political belief in household response to health risks. Results further underscore the importance of timely, targeted messaging and intervention among divergent political belief groups to reduce vaccine hesitancy and enhance disease control. Future studies should explore the external validity of findings, including the use of individual voter data, if available, to evaluate political belief effects.

## 1. Introduction

Substantial anecdotal evidence and survey-based research suggest the salience of household worldview and political belief to COVID-19 vaccine resistance, virus transmission, and efficacy of policy treatment. Pandemic-era reports indicate striking disparities among belief groups in disease propagation as well as related challenges to government policymakers seeking to lift vaccine uptake and mitigate virus spread. For example, a Kaiser Family Foundation COVID-19 Vaccine Monitor survey published on 2 December 2021 reported that 90 percent of Democrats had received at least one COVID-19 vaccination, compared with 60 percent of Republicans. Further, a 2021 Pew Research Center survey similarly reported that 45 percent of the 41 million white Christian evangelicals in the U.S. were vaccine resistant (see New York Times, “White Evangelical Resistance Is Obstacle in Vaccination Effort”, 5 April 2021). Survey-based literature on political worldview and COVID-19 includes papers on perception of virus threat [1,2], willingness to get vaccinated [3,4,5,6,7,8,9], compliance with social distancing [1,10,11], mask-wearing [1,12], and other guidelines [3].

While survey research has importantly contributed to our understanding of associations between politics and COVID-19 risk-related behaviors, there has been little well-controlled estimation or related simulation of the effects of political belief on *actual* COVID-19 vaccination behavior and virus transmission. Additionally, we know little about whether belief-related COVID-19 behaviors were durable or evolved in the wake of exposure to virus risk. Also, there is limited understanding of how political belief affected the success of major COVID-19 mitigation efforts, notably including economic closure. Such information is critical to the specification of effective disease control policies and, more generally, to the understanding of the role of political belief in response to prevailing health risks. Arguably, ideology and political belief remain highly salient to vaccine resistance, virus transmission, and compliance with public health directives.

The purpose of this study is to examine the role of political belief in COVID-19 vaccine uptake, virus transmission, and closure policy response. We employ unique panel data on all COVID-19 vaccination and infection cases in Israel over the March 2020–April 2021 study period. Model estimates are used to simulate the aggregate effects of more risk-averse political beliefs on vaccine resistance and disease transmission. Israel provides an ideal laboratory to analyze the role of political belief in COVID-19 virus risk, vaccination resistance, and related policy treatment. The country is comprised of diverse populations holding significantly divergent worldviews and political beliefs, including left- and right-wing ideologues, Arab ethnic and religious minorities, and orthodox religious Jewish groups. Moreover, COVID-19 policy interventions in Israel were not framed in partisan terms and were broadly supported by leaders across the political spectrum. Hence, unlike the U.S. and elsewhere, household response to COVID-19 risk in Israel was not biased by contemporaneous disagreement and partisan debate among political leaders. (for analyses of partisan disagreement and COVID-19 health behaviors in the U.S., see [13,14,15]). Additionally, the provision of universal health care and related availability of comprehensive electronic medical records in Israel allowed full, accurate, and timely tracking of virus incidence and vaccine uptake. Israel was an early adaptor of comprehensive testing and vaccination but experienced three severe spikes in virus incidence over the course of our study period. As of February 2021, Israel led the world with a population first-dose vaccination rate of 28 percent, more than three times the next highest country. By the end of our sample (April 2021), Israel had a first-dose vaccination rate of 58 percent and was ranked first in the world [see https://ourworldindata.org/covid-vaccinations (accessed on 17 March 2022)].

We characterize political belief based on statistical area voting outcomes in Israel parliamentary (Knesset) elections held shortly before the COVID-19 outbreak in March 2020. Statistical areas in Israel are defined to include 3000–5000 residents and are roughly equivalent to U.S. census tracts (see [16]). Those areas were exogenously determined prior to the 2020 COVID-19 pandemic by the Israel Central Bureau of Statistics. We combine that information with extensive statistical area population socio-economic, demographic, housing, geographic access, and civic engagement characteristics. Results of panel estimation indicate substantial variation in COVID-19 vaccine uptake, virus transmission, and closure policy response among areas characterized by divergent beliefs. All things equal, findings show that when confronted by emergent and localized virus infection risk, residents of politically right-of-center and religious areas were associated with substantially higher odds of both vaccine resistance and virus transmission compared to their left-center counterparts. For purposes of assessing the robustness of infections and vaccinations to the virus testing regime, we also conducted all statistical tests using COVID-19 hospitalization outcomes. Results were generally robust to hospitalizations (as described below, we include those findings in Appendix A).

Model simulation shows that political belief was highly salient to aggregate pandemic outcomes. Specifically, had populations in all areas responded to virus risk with the more risk-averse behaviors of left-of-center areas, the number of vaccinations nationwide would have increased by 15 percent. That same scenario results in a full 30 percent reduction in total infection cases. Finally, we find that more coercive economic closure policies imposed by the Israeli Government in September–October 2020 in response to the virus surge were more effective in abating virus risk among right-of-center and religious areas characterized by less risk-averse behaviors.

Our findings make a number of contributions to the literature. First, unlike prior survey reports, we provide systematic and well-controlled analyses of the role of political belief as mediated by emergent COVID-19 disease risk on pandemic outcomes, including vaccine uptake, virus transmission, and response to closure policy treatment. Further, unlike many other countries, COVID-19 response and policy treatment in Israel were not the subject of acrimonious and well-publicized partisan leader disagreement; as such, our estimated effects of political belief are not biased by leadership politicization of COVID-19 health behaviors and policy. Additionally, unlike previous studies, we simulate the substantial reduction in aggregate vaccine resistance and virus transmission associated with the more risk-averse political beliefs of left-center areas. Results provide new evidence of the durability of political belief and related behaviors in the face of palpable health risks.

## 2. Materials and Methods

### 2.1. Data

We identify households that ex ante likely hold divergent political beliefs based on voting outcomes in 1350 small statistical areas (akin to census tracts) in general (parliamentary) elections held in Israel in March 2020 and just prior to the COVID-19 outbreak (Israel Central Elections Committee). One of the strengths of the Israeli pre-pandemic voting data is that they are not contaminated by COVID-era policy disagreement and hence provide an indication of how underlying political belief affects disease behaviors and outcomes (vaccine resistance, infections, hospitalizations, and response to closure policy). The use of small-area voting data to proxy political belief is well-vetted in the published literature (e.g., Refs. [14,15,17]). Those papers employ similar measures, including county-level votes in U.S. presidential elections, to document partisan bias associated with public health, financial, and economic outcomes. We merge this information with a weekly panel of all vaccination and infection cases in Israel from the outbreak of COVID-19 in March 2020 through the end of April 2021 (Israel Ministry of Health—IMF), as well as statistical area controls for population socio-economic, demographic, housing, and geographic access (Israel Central Bureau of Statistics). In 36 of the 1386 statistical areas in the IMF dataset, there was an error in the number of weekly vaccinations. After consulting with the IMF, these were removed from our panel.

Table 1 provides summary information on statistical area controls. As shown, the average number of persons per statistical area (Pop) is 4,589, and the average population density (Density) is 13,177 persons per square kilometer. The population is relatively young and characterized by high birth rates: the average share of the Israeli population over the age of 60 (Age60) is 0.20, whereas the average share of the population under the age of 15 (Age15) is 0.24. We use the ICBS (2013) socio-economic index score (SES) to control for statistical area variation in household income and education. The socio-economic index is computed based on 16 indicators clustered into four groups: standard of living, employment and welfare, schooling and education, and demography. The 16 indicators include average years of education for the population age 25–54; share of the population with academic degree age 25–54; share of workers in academic or management positions; share of income earners age 15 and above; share of women age 25–54 not in the workforce; share of workers on the job at least two days per week; share of income earners below minimum wage; share of the population with income support; average per capita income; the average number of cars per household; the average number of rooms per household; the average number of bathrooms per household; share of households with computer and internet connection; median age, dependency ratio, and the average number of persons per household. The socio-economic index is generated by factor analysis that reduces the 16 indicators to three main factors that explain 80% of the variation among the statistical areas (see [18]). As shown, the average socio-economic index score is about 0.22, with a standard deviation of 1.01. We also control for the geographical proximity of the statistical area to Tel Aviv, the “superstar” city and central business district of Israel (see, e.g., Ref. [19]). The table also provides information on the share of non-voters among the eligible local voting population (NonVoter), which proxies for reduced civic engagement and social capital (e.g., Refs. [20,21,22,23]) as may adversely affect vaccine uptake and response to policy treatment.

Table 1 also presents summary statistics for statistical areas clustered by distinct ideological worldviews and political beliefs. We proxy for statistical area political belief using the distribution of votes among political parties in Israel’s March 2020 national parliament elections. We compute votes by the party in each of the statistical areas and then use the k-means clustering method (see [24,25]) to classify each of the 1350 statistical areas into one of five political belief groups. Essentially, the k-means procedure partitions N observations into k sets, minimizing the within-set variance. The k number of sets is determined based on the elbow method (see, e.g., Ref. [26]).

**Figure 1:**
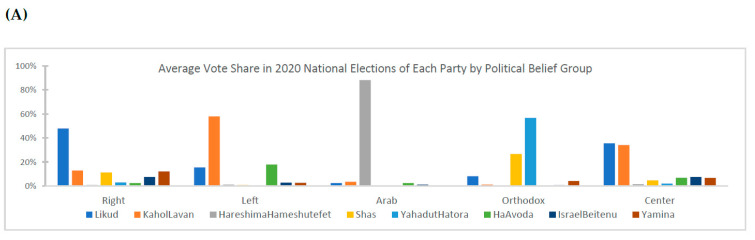

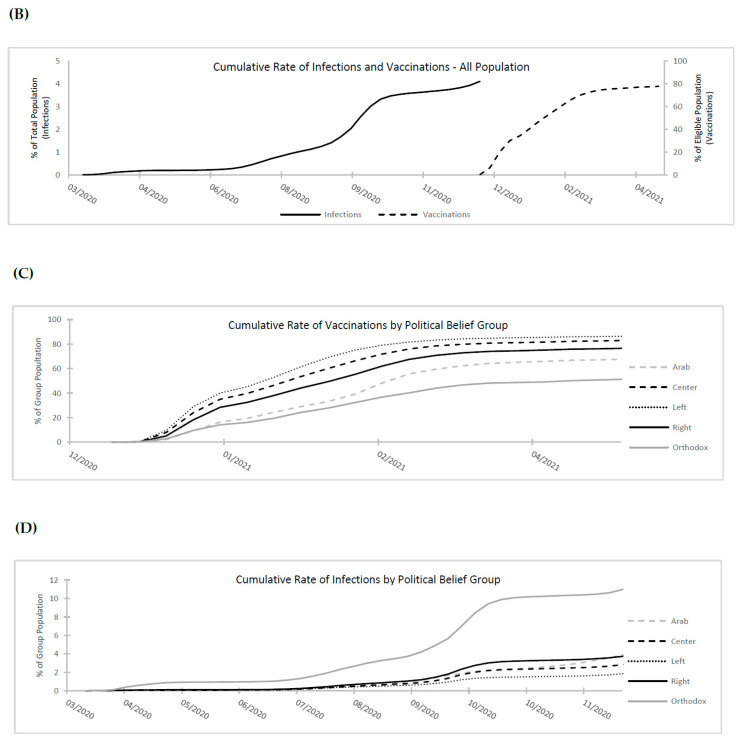
Cumulative rates of COVID-19 infections and vaccinations (total and by political belief group) and average vote rate for political parties by political groups. Notes: Figure (**A**) shows the average vote share in the 2020 national elections of each party by political worldview and belief; Figure (**B**) shows cumulative rates of COVID-19 vaccinations and infections for the entire population; Figure (**C**) shows cumulative rates of vaccination by belief group; and Figure (**D**) shows cumulative rates of infection by belief group.

Panel A of Figure 1 presents the average political party vote share by belief group, including Right, dominated by votes for “Likud” and “Yamina” (38 percent of statistical areas in the sample); Left, reflecting a high share of votes for “Kahol-Lavan” and “HaAvoda-Meretz” (19 percent); Center, characterized by roughly equivalent votes for “Likud” and “Kahol-Lavan” (28 percent); the non-Jewish Arab minority, as defined by a high share of votes for the united Arab list “Hareshima Hameshutefet” (5 percent); and the highly observant Jewish religious Orthodox, dominated by votes for “Yahadut Hatora” and “Shas” (10 percent). In Israel, the Orthodox political parties represent the priorities and imperatives of highly observant Orthodox Jewish voters, notably including the closure of a broad range of places in observance of the Jewish Sabbath, funding of religious seminary students and institutions, limitations on military conscription, and so on. Following Panel A in Figure 1, we label the political belief groups by Right, Center, Left, Arab, and Orthodox based on their respective vote share. Accordingly, as shown in Table 1, Left areas, on average, exhibit the highest socio-economic index score, the lowest housing density, and are closest to Tel Aviv. In contrast, Orthodox statistical areas exhibit the highest housing density and household size, the youngest population, and the lowest average socio-economic index score.

### 2.2. The Model

To identify the effects of political belief on COVID-19 vaccinations and infections, we comprise a weekly panel among the 1350 statistical areas and estimate the following model:

(1) $$Y_{it}=\beta_{0}+{\vec{\beta }}_{1}I_{i}+\beta_{2}Infections_{i,t-1}+{\vec{\beta }}_{3}I_{i}\times Infections_{i,t-1}+{\vec{\beta }}_{4}X_{i}+{\vec{\beta }}_{5}\tau_{t}+\varepsilon_{1it},$$

where the outcome term $Y_{it}$ is the log odds (i.e., $\ln\left[p_{it}/\left(1-p_{it}\right)\right]$) of either first-dose vaccination uptake or infection in location (statistical area) *i* at the time (week) *t* and where $p_{it}$ is the probability of vaccination uptake (infection), computed as the ratio of vaccinations (infections) to eligible (at risk) population for all *i* and *t*. Note that the eligible population for vaccination changes over time in accordance with public health protocol that allows vaccination of increasingly younger age groups. Additionally, we subtract those already vaccinated from the eligible vaccination population. As pertinent to the timeframe of this analysis, we further assume that those already infected are not subject to infection risk; thus, the population at risk of infection declines over time. Over the timeframe of our analysis, Ref. [27] found that recovery from COVID-19 largely provided immunity to virus re-infection from variants Alpha, Beta, and Gamma.

The vector I in Equation (1) represents a series of political belief fixed-effects based on the k-means classification procedure (described above), including Right (base category); Left; Center; Arab; and Orthodox areas. Other controls include statistical area virus incidence as measured by the log of the number of COVID-19 infection cases in the prior week, $Infections_{t-1}$; interactions of the vector I with $Infections_{\;t-1}$, which enables estimation of response by political belief to local virus risk as proxied by the lagged number of statistical area weekly infections (where $\mathrm{Right}\times Infections_{\;t-1}$ is the base category); and X, a vector of statistical area characteristics including Pop, the population size of the statistical area; Density, the ratio between the number of people in the statistical area and the geographic size in square meters; Age60, the share of the population over the age of 60; Age15, the share of population under the age of 15; PersonHH, the average number of persons per household; RoomsHH, the average number of rooms per standard person; ProximityTA, the standardized proximity of the statistical area from Tel Aviv; NonVoter, the share of non-voters among the population eligible for voting in the statistical area; and SES, the socio-economic index score of the statistical area. Finally, the estimating equations include a vector τ of time (week) fixed-effects, $\beta_{0}$ and $\beta_{2}$ are estimated parameters, ${\vec{\beta }}_{1}$ and ${\vec{\beta }}_{3}-{\vec{\beta }}_{5}$ are vectors of estimated parameters, and $\varepsilon_{1}$ is a random disturbance term. Following [14,15] and survey-based research (among others, [1,3]), we expect that the coefficients comprising the vector ${\vec{\beta }}_{4}$ are significantly different from one another, reflecting varying vaccination and infection behavior across belief groups in response to local virus risk.

## 3. Results

As shown in Panel B of Figure 1, over successive pandemic virus waves through the end of December 2020, about 4.1 percent of the Israeli population was infected with COVID-19. Additionally, following the commencement of the vaccination campaign in December 2020–April 2021, about 78 percent of the eligible population aged 16 and over received at least one vaccine dose (only the Pfizer vaccine was available in Israel). Panels C–D in Figure 1 show salient differences in cumulative virus infection and vaccination rates over the sample period and among political belief groups. Summary information indicates elevated infection rates and low vaccination uptake among orthodox Jewish and, to a lesser degree, right-leaning and Arab areas, whereas the left-leaning group exhibited the highest (lowest) uncontrolled rate of vaccinations (infections).

We report results from the estimation of Equation (1) separately for COVID-19 vaccination and infection outcome terms. We use the weighted least squares procedure in all estimations, whereby the weight is determined by eligible populations (respectively for vaccinations and infections) in *i* and *t*. Additionally, we assess the robustness of belief findings to continuous versions of those controls and replacement of the one-week lagged infections term with lagged hospitalizations and two-week lagged infections. As described below, the estimated belief effects are largely robust to those model specifications. Hence those results are relegated to Appendix A. In the statistical analysis, we used Anaconda in conjunction with Python 3.6.8. along with the following packages: geopandas 0.6.1; linearmodels 4.18; numpy 1.19.3; pandas 1.1.3; scikit-learn 0.21.2; and scipy 1.2.1.

### 3.1. Vaccinations

Table 2 presents the results of panel estimation of Equation (1) on the log odds of first-dose vaccination among 1350 statistical areas (of the about 1650 statistical areas in Israel) over the 19 weeks from the commencement of the vaccination campaign on 20 December 2020 through 25 April 2021. Full results from the estimation of log odds of first-dose vaccination, inclusive of control terms, appear in Appendix A Table A1. We ended the weekly vaccination sample on 25 April 2021 as the number of daily doses per capita and infection cases per 1 million persons dropped to 0.12 and 5, respectively. At that time, about 78 percent of the eligible population at the age of 16 and over had received at least one dose of the Pfizer vaccine. Column 1 presents benchmark outcomes controlling only for political belief group fixed-effects (vector I; Right serves as the base group). As shown, statistical areas characterized by Orthodox beliefs exhibit the lowest likelihood of vaccination uptake, followed by Arab, Right, Center, and Left. The estimated belief coefficients are significantly different from one another at the 1 percent level with the exception of the insignificant difference between the coefficients for Right and Arab.

In column 2, we re-estimate that model, including the controls (described above) exclusive of the lagged infection terms. Results here differ from both uncontrolled estimates (column 1 and Figure 1 Panel C) and from findings of the survey-based literature (discussed earlier). Specifically, controlling for socio-economic status, population density, civic engagement as proxied by share of non-voters, age distribution, housing, and other factors, the effect of political belief on the likelihood of vaccine uptake (column 2) is insignificantly different among areas characterized by divergent political beliefs. However, this specification fails to control for local infection risk.

In column 3 of Table 2, we evaluate the extent to which the estimated effects of political belief on vaccine uptake are mediated by the risk of exposure to COVID-19 as proxied by local infection risk. We do so by including $Infections_{t-1}$ and the vector of interaction terms $I\times Infections_{t-1}$ on the right-hand side of the log odds of vaccination Equation (1) (together with other socio-economic and demographic controls). Summing the coefficients on $Infections_{t-1}$ and the interaction term $I\times Infections_{t-1}$ indicates that ceteris paribus, a 1 percent increase in the weekly lagged number of local infection cases is associated with a 0.52, 0.32, 0.28, 0.17, and 0.12 percent increase in the odds of vaccination take-up among statistical areas characterized by Left, Arab, Center, Right, and Orthodox worldviews, respectively (all significant at the 1 percent level). Additionally, the interactive political belief effect coefficients (associated with the vector $I\times Infections_{t-1}$) are largely different from one another at the 1 or 5 percent level (with the exception of the coefficients for Center and Arab).

**Figure 2:**
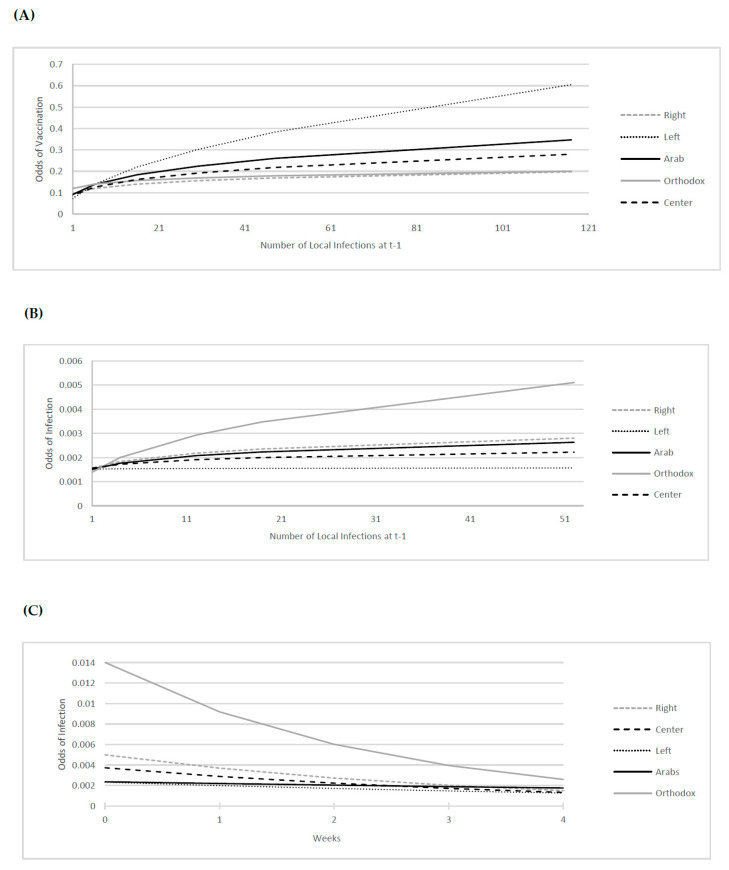
Estimated Belief Group Odds of Vaccination and Infection by Lagged Infections (Panels (**A**,**B**), respectively) and Odds of Infection Response to Policy Closure (Panel (**C**)). Notes: Figure (**A**,**B**), respectively, present estimated political belief group average vaccination and infection odds by $Infections_{t-1}$, where the latter ranges from the 1st to the 99th percentile of its sample distribution. Figure (**C**) presents the sum ${\bar{Y}}_{i0}I_{i}+{\hat{\gamma }}_{2}t+{\vec{\hat{\gamma }}}_{3}I_{i}\times t$ for all *i* and *t* = 0, 1, …, 4 from estimates in column 8 of Table 2—in odds terms—where ${\bar{Y}}_{i0}I_{i}$ is the group average odds of infections at the beginning of the closure.

Panel A in Figure 2 depicts the projected odds of vaccination uptake associated with 1-week lagged infection risk by political belief (the exponent of the sum of ${\hat{\beta }}_{0}+{\vec{\hat{\beta }}}_{1}I+{\hat{\beta }}_{2}Infections_{t-1}+{\vec{\hat{\beta }}}_{3}I\times Infections_{t-1}$ for all I—holding all other control terms equal to zero), where $Infections_{t-1}$ ranges from the 1^st^ to the 99th percentile of its sample distribution (over the period December–April 2021). All things equal, while areas on the Left exhibit a damped rate of vaccination uptake for low levels of health risk ($Infections_{t-1}$), vaccination response among those areas rises as local health risk increases. In marked contrast, conservative areas holding Orthodox and Right political beliefs appear largely impervious to localized and immediate COVID-19 infection risk. As such, Left and Orthodox/Right areas represent two ends of a response (to health risk) distribution; in the former case, the initial low level of vaccine uptake is mediated and informed by increasingly elevated disease risk to improve vaccination response, whereas the opposite finding is evidenced in the case of areas holding conservative beliefs. Indeed, areas holding Orthodox and Right worldviews demonstrate damped responsiveness in vaccine uptake even when confronted by ever-increasing local infection risk, suggesting related challenges to vaccination campaigns in the management and control of the pandemic. These findings are consistent with survey reports on the association between political conservativism and vaccine resistance (e.g., Refs. [4,5,6,7,8]).

Note that the estimated vector of $I\times Infections_{t-1}$ political belief interaction terms is robust to the inclusion of a full set of interactions of $Infections_{t-1}$ with socio-economic, age, and density controls. Specifically, we supplement the right-hand side of the log odds of vaccination equation with interactions of $Infections_{t-1}$ with SES, Age60, Age15, and Density. Results (not presented but available upon request) are robust to this model specification. Further, results throughout are largely robust to the continuous specification of belief terms and the replacement of $Infections_{t-1}$ with either $Hospitalizations_{t-1}$ or $Infections_{t-2}$. Results of estimation of continuous belief terms as well as hospitalization and lagged infection models are presented in Table A2 and Table A3 of Appendix A. Note further that among controls (Columns 2–3 in Table A1), socio-economic status index (SES) is positively associated with odds of vaccine uptake (significant at the 1 percent level), whereas the coefficient on NonVoter implies that a 1 basis point increase in the share of non-voters among the eligible population is associated with 1.2–1.5 percent reduction in the odds of vaccination incidence (significant at the 1 percent level). As in [21], who find that civic capital is associated with compliance to social distancing, our results suggest that political disengagement or disaffection among the local population may adversely affect the success of vaccination campaigns.

### 3.2. Infections

In columns 4–6 of Table 2, we repeat the panel estimation of Equation (1), replacing the dependent variable log odds of statistical area weekly vaccinations with the log odds of weekly COVID-19 virus infections for March–December 2020. The analysis of infection cases ends on 20 December 2020 due to the commencement of vaccinations and detection of the more contagious Alpha and Beta variants in Israel. As described above, the odds of infection during the 15 March–20 December 2020 sample are computed as the ratio of infection cases to all uninfected populations for all statistical areas *i* and weeks *t*. Empirical findings provide evidence of the salient effects of political belief on virus propagation. Specifically, in column 4, we include only belief fixed effects (i.e., vector I; Right serves as the base group). As shown, statistical areas in the Left group exhibit the lowest average infection likelihood, followed by the Center, Right, Arab, and Orthodox belief groups. In column 5, we include the full set of controls exclusive of the lagged local infection terms (the results on the socio-economic and demographic controls from the estimation of the log odds of infection Equation (1) appear in Appendix A Table A1). In both columns 4 and 5, the political belief fixed effects coefficients are generally different from one another at the 1 percent level. The exceptions here are the couplets Arab/Right in column 4 and Arab/Left and Arab/Center in column 5, which are insignificantly different from one another. While the pattern of belief effects on disease incidence, as shown in column 5, is generally similar to that shown in column 4, the estimated magnitudes are damped upon the inclusion of controls.

In column 6, we assess the extent to which the estimated effects of political belief on virus transmission are mediated by exposure to infection risk, as proxied by lagged statistical area infection cases. As mentioned above, we include $Infections_{t-1}$ and the vector of $I\times Infections_{t-1}$ interaction terms. As shown, ceteris paribus, statistical areas on the Left are associated with the lowest likelihood of disease transmission in response to lagged infection cases. Summing the coefficients on $Infections_{t-1}$ and the interaction term $I\times Infections_{t-1}$, a 1 percent increase in the number of weekly lagged infections is associated with a 0.01, 0.11, 0.17, 0.18, and 0.40 percent rise in the odds of infection among areas holding Left, Center, Arab, Right, and Orthodox views, respectively (all significant at the 1 percent level). Additionally, the belief and infection incidence interactive coefficients (associated with the vector $I\times Infections_{t-1}$) are different from one another at the 1 percent level (except for the insignificant difference between the Arab/Right pair). 

Panel B in Figure 2 plots the projected odds of infection associated with the political belief by 1-week lagged local infection risk (sum of ${\hat{\beta }}_{0}+{\vec{\hat{\beta }}}_{1}I+{\hat{\beta }}_{2}Infections_{t-1}+{\vec{\hat{\beta }}}_{3}I\times Infections_{t-1}$ for all I—holding other controls equal to zero), where $Infections_{t-1}$ ranges from the 1st to the 99th percentile of the sample distribution (over March–December 2020). As shown, at low levels of infection, ceteris paribus, there is little difference in infection propagation by political belief. Further, the odds of infection inevitably rise over the course of the pandemic regardless of political belief. That said, statistical areas on the Left exhibit the lowest disease transmission in response to increased exposure to the infection risk, followed by those holding Center, Arab, Right, and Orthodox beliefs. All else equal, while infection risk among the Left is much informed by increasing localized exposure to disease, such is not the case among areas characterized by conservative Orthodox and (to a somewhat lesser extent) Right beliefs. In Orthodox and Right areas, we find a sharply elevated likelihood of disease transmission as infection rates rise, suggesting damped responsiveness among those holding conservative beliefs even when confronted by growing and immediate local health risks.

In response to the virus surge, national economic closure was imposed by the Israeli Government during the 4 April–4 May 2020 and 25 September–17 October 2020 periods. We assess the robustness of estimated interactive political belief and lagged infection results for the period between closures (10 May–24 September 2020). Outcomes (column 7 of Table 2) are generally robust across the full and sub-sample periods, as the sums of the coefficients on $Infections_{t-1}$ and $I\times Infections_{t-1}$ for all I are insignificantly different from one another across the two samples. Additionally, as noted earlier, the estimated $I\times Infections_{t-1}$ belief interaction terms are robust to (*a*) the inclusion of a full set of interactions of $Infections_{t-1}$ with SES, Age60, Age15, and Density controls (*b*) continuous specification of political belief terms (see Table A2 in Appendix A); and (*c*) the replacement of $Infections_{t-1}$ with either $Hospitalizations_{t-1}$ or $Infections_{t-2}$ (see Table A3 in Appendix A). Finally, note that among the controls (columns 5–6 in Table A1), the coefficient on socio-economic status (SES) is negative and significantly associated with the odds of infection.

### 3.3. Costs of Risky Behavior

Differences in response to health risks among political groups carry a social cost. As estimated above, political groups associated with risky COVID-19 behaviors impose higher costs on society via lower rates of vaccination and higher disease incidence. In this section, we assess the aggregate COVID-19 costs of less cautious responses to virus risk. Specifically, we employ results of model estimation to simulate how nationwide vaccinations and virus infections would have changed had all statistical areas responded to virus risk, as did more risk-averse left-of-center areas. Per the results of the estimation of Equation (1), recall that other areas, including those supporting Center, Right, Arab, and Orthodox political parties, exhibited significantly lower odds of vaccination and higher infection odds upon exposure to COVID-19 virus risk.

Recall that the Left areas are most likely to get vaccinated in the face of virus risk. To simulate the effect of divergence in political beliefs on vaccination uptake by belief group, we project ${\hat{Y}}_{it}=\hat{\ln\left[p_{it}/\left(1-p_{it}\right)\right]}$, the log odds of vaccination uptake in the statistical area *i* at week *t*, by substituting in place of the estimated coefficient for each belief group that of the Left on the right-hand side of Equation (1) (see results in column 3 of Table 2). We then extract ${\hat{p}}_{it}$, the projected probability of vaccination uptake for all *i* and *t* had area *i* behaved as Left, and compute the proportion ${\hat{p}}_{it}/p_{it}$, where $p_{it}$ is the actual probability of vaccination uptake in area *i* at time *t*. Multiplying the ratio ${\hat{p}}_{it}/p_{it}$ by the actual number of vaccination cases in *i* and *t* generates the projected number of vaccination uptakes in *i* and *t*, had area *i* behaved as Left. In simulating the effect of political belief divergence on vaccination uptake, we control for the actual lagged number of infections on the right-hand side of Equation (1). We conducted this simulation among 1350 statistical areas over the 19 weeks from the commencement of the vaccination campaign from 20 December 2020 through 25 April 2021.

**Figure 3:**
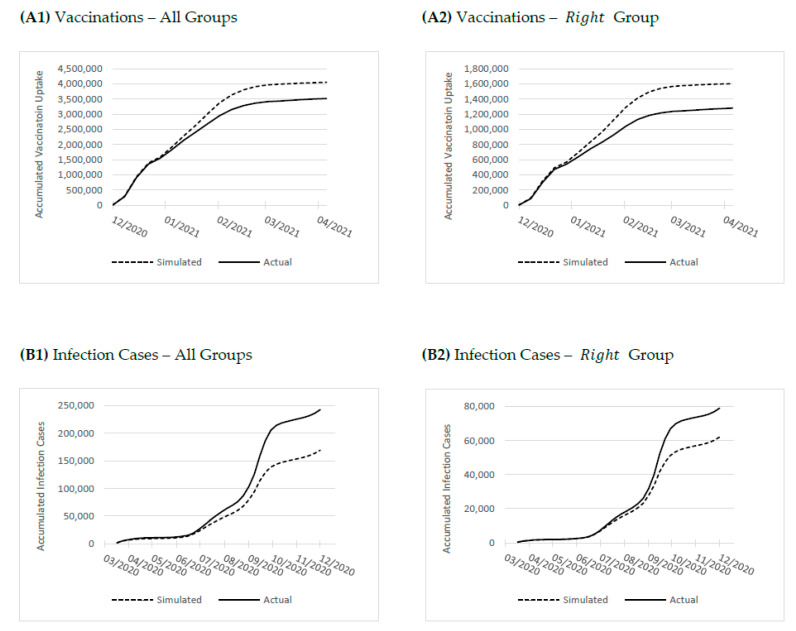
Simulated (versus actual) infection incidence and vaccination uptake had political belief groups responded to virus risk with behaviors of the Left group.

Exhibit A1 in Figure 3 presents simulation outcomes of accumulated projected vaccination uptake across all areas had they instead uniformly adopted the vaccination behavior of the Left. As shown, by the end of April 2021, some four months into the vaccination campaign, the total number of first-dose vaccinations across all statistical areas would have increased by about 15 percent (from 3.51 to about 4.05 million). Among belief groups, had politically-Right areas assumed the vaccination behavior of the Left, the number of first-dose vaccination would have increased by more than 25 percent (from about 1.28 to about 1.60 million—see Exhibit A2 in Figure 3).

We similarly quantify the effect of less risk-averse political beliefs on the aggregate number of infection cases. We project ${\hat{Y}}_{it}=\hat{\ln\left[p_{it}/\left(1-p_{it}\right)\right]}$, the log odds of infection in the statistical area *i* at week *t*, substituting the dummy variable coefficient of each political belief group with that of the Left on the right-hand side of Equation (1) (see results in column 6 of Table 2). We then extract ${\hat{p}}_{it}$, the projected probability of infection for all *i* and *t* had residents of the area *i* adopted Left pandemic behaviors and computed the ratio ${\hat{p}}_{it}/p_{it}$, where $p_{it}$ is the actual probability of infection in area *i* at time *t*. Multiplying the ratio ${\hat{p}}_{it}/p_{it}$ by the actual number of infection cases in *i* and *t* generates the projected number of infection cases in *i* and *t*, had area *i* engaged in the more cautious COVID-19 behaviors of the Left. Finally, as the term $Infections_{i,t-1}$, the log of the number of COVID-19 infection cases in the prior week, appears on the right-hand side of Equation (1), we replace $Infections_{i,t-1}$, the actual lagged number of infection cases, with ${\hat{Infections}}_{i,t-1}$, the projected lagged number of infection cases, for all *i* and *t* based on the above described procedure in computing ${\hat{Y}}_{it}$. As in our primary specification above, we conduct this simulation for 1350 statistical areas over March–December 2020.

Exhibit B1 in Figure 3 presents the simulated cumulative infection incidence, had all areas responded to virus risk with behaviors akin to those of the Left. As shown, divergence in political belief is highly salient to aggregate disease outcomes. Indeed, the total number of infection cases for the March–December 2020 period would have dropped by a full 30 percent (from about 242,000 to about 169,000) had all areas responded to virus risk with the same risk-averse behaviors of left-of-center areas. Specifically, among areas, had those on the Right adopted the behaviors of the Left in response to virus risk, the number of infection cases in Right statistical areas would have dropped by about 22 percent (from about 79,000 to about 62,000—see Exhibit B2 in Figure 3).

### 3.4. Event Study: Political Belief Response to COVID-19 Closure

In the wake of limited household COVID-19 risk aversion and high prevailing levels of virus incidence, policymakers may require more stringent measures of disease control. Akin to the above simulations, such interventions seek to impose consistent disease practices on disparate belief groups. In this section, we examine treatment outcomes associated with the country-wide closure imposed by the Israeli Government in response to the virus surge during 25 September–17 October 2020. While this date represents the official timeframe of the closure, entrance to and exit from closure was gradual. During the lockdown period, national virus-related restrictions were implemented, including a stay-at-home order; a shutdown of schools, universities, and non-essential retail and workplaces; and only limited provision of public transportation. The population limitations imposed before, during, and after the closure were identical across statistical areas. Additionally, Israel imposed two other monthly closures in April 2020 and January 2021. We omit assessment of behavioral response to those closures, as the former was associated with low morbidity rates, and the latter reflects in part evolution in both the virus itself (increased prevalence of Alpha and Beta variants in Israel) and in vaccination take-up.

To assess closure policy treatment response across areas characterized by political belief, we estimate the following COVID-19 infection odds equation:

(2) $$Y_{it}=\gamma_{0}+{\vec{\gamma }}_{1}I_{i}+\gamma_{2}Infections_{i,t-1}+{\vec{\gamma }}_{3}I_{i}\times Infections_{i,t-1}+\gamma_{4}t+{\vec{\gamma }}_{5}I_{i}\times t+{\vec{\gamma }}_{6}X_{i}+\varepsilon_{2it},\;$$

where the dependent variable, $Y_{it}$, is the log odds of infection in week *t* and statistical area *i*. The estimation of Equation (2) differs from that of Equation (1) in two ways. First, we estimate Equation (2) only for the closure period and restrict the sample for weeks *t* = (0, 1, …, 4), where *t* = 0 is the week when the closure commences. Further, we omit τ (weekly fixed-effects) and supplement Equation (2) with the vector $I_{i}\times t$, a series of interaction terms between the political belief fixed-effect and a time trend, to estimate divergent infection response paths to closure by belief group. Additionally, $\gamma_{0}$, $\gamma_{2}$, and $\gamma_{4}$ are estimated parameters, ${\vec{\gamma }}_{1}$, ${\vec{\gamma }}_{3}$, and ${\vec{\gamma }}_{5}-{\vec{\gamma }}_{6}$ are vectors of estimated parameters, $\varepsilon_{2}$ is a random disturbance term, and all other variables are as discussed above.

Column 8 in Table 2 presents the results of the event study panel estimation of the infections model for the closure treatment period. Consistent with the outcomes in the previous section, response to lagged infections (i.e., the sums of the coefficients on $Infections_{t-1}$ and $I\times Infections_{t-1}$) varies by the political belief of the statistical area. As above, Orthodox areas, ceteris paribus, exhibit the highest odds of disease transmission in response to lagged infection risk, followed by those characterized by Arab, Right, Center, and Left worldviews. The estimated difference between each pair of belief coefficients is significant at the 1 percent level with the exception of the Arab/Right and Arab/Center pairs, which are insignificantly different from one another.

Moreover, while results indicate that the pandemic economic lockdown was effective in decreasing the likelihood of infection cases among all areas (as the sum of coefficients on t and the vector $I_{i}\times t$ are all negative and significant at the 1 percent level), the rate of decline in the likelihood of infections during closure varied by area political belief. Specifically, each additional week of closure was associated with an average of 0.42, 0.30, 0.26, 0.15, and 0.07 percent decrease in odds of infection among areas holding Orthodox, Right, Center, Left, and Arab views, respectively (all significant at the 1 percent level). The estimated response to government-imposed closure by political belief is plotted in Panel C of Figure 2. The plots compute the sum ${\bar{Y}}_{i0}I_{i}+{\hat{\gamma }}_{2}t+{\vec{\hat{\gamma }}}_{3}I_{i}\times t$ for all I and *t* = 0, 1, …, 4 as follows from the estimation results in column 8 of Table 2—translated to odds terms—where ${\bar{Y}}_{i0}I_{i}$ is the political belief group’s average odds of infections (across statistical areas) at the beginning of the closure. As shown, the decline in infection odds during closure was most precipitous among Orthodox areas, followed by those characterized by Right, Center, Left, and Arab beliefs. Further, the pair-wise difference between any pair of beliefs in the decline in odds of infection during closure is significant at the 1 percent level. Findings of heterogeneity in closure effects among areas characterized by political belief are generally robust to continuous specification of the belief effects (see Table A2 in Appendix A); and to the replacement of $Infections_{t-1}$ with either $Hospitalizations_{t-1}$ or $Infections_{t-2}$ (see Table A3 in Appendix A). In sum, results suggest that imposition of state-mandated closure policy treatment was most effective in imposing virus mitigation behaviors on conservative (Orthodox and Right) areas most likely to become infected and to resist vaccination in response to infection risk.

## 4. Discussion

Previous survey-based studies have found evidence of an association between political worldview and vaccine resistance. COVID-19 further brought this issue to the forefront as governments around the world struggled to promote vaccination uptake and pandemic disease control. In this study, we use data from Israel on 2020 statistical area general election voting outcomes, panel information on the universe of all COVID-19 vaccinations and virus transmissions, and numerous local area socio-economic and other controls to assess the role of political beliefs in COVID-19 vaccine resistance and virus outcomes. COVID-19 policy interventions in Israel were not framed in partisan terms and were broadly supported by leaders across the political spectrum. Hence, unlike the U.S. and elsewhere, household response to COVID-19 risk in Israel was not biased by contemporaneous disagreement and partisan debate among political leaders. As discussed below, findings show that political belief is highly salient to COVID-19 vaccine resistance, virus transmission, and response to national closure policy.

Upon controlling for socio-economic and demographic characteristics, we estimated belief effects on vaccine resistance and virus transmission and further assessed whether those estimates were mediated by exposure to immediate and localized infection risk. Our findings show that vaccination take-up in response to 1-week lagged local virus incidence was lowest among Orthodox/Right areas, followed by those characterized by Center, Arab, and Left beliefs. Similarly, as expected, the COVID-19 infection odds associated with 1-week lagged local virus incidence were highest for areas with Orthodox/Right worldviews, followed by Arab, Center, and Left beliefs. These findings provide new evidence of salient behavioral differences among areas characterized by divergent political beliefs in the transmission of and vaccination response to immediate COVID-19 disease risk. Overall, our evidence shows that upon exposure to local virus risk, statistical areas associated with conservative beliefs, as compared to liberal areas, are associated, ceteris paribus, with greater vaccine resistance and higher odds of virus transmission. These findings are consistent with and deepen insights surrounding survey-based associations between political conservativism and vaccine resistance (e.g., Refs. [4,5,6,7,8]).

Results show that political differences in COVID-19 risk response may carry a substantial social cost. We simulate the estimated model to assess how the aggregate number of vaccinations and infections would have changed had all areas responded to virus risk with the more risk-averse behaviors of left-of-center areas. Indeed, we find that had all areas adopted the vaccination behavior of left-to-center neighborhoods, the total number of first-dose vaccinations would have increased by about 15 percent; further, among the Right, vaccinations would have increased by more than 25 percent. That same simulation would have served to reduce total infection cases by a full 30 percent. Our simulation thus quantifies the substantial health costs associated with divergent political beliefs in the face of emergent health risks.

Finally, our findings show a heterogenous response among areas characterized by divergent political beliefs to country-wide closure imposed in the wake of the COVID-19 virus surge in September–October 2020. Results indicate that while the lockdown was effective in decreasing the odds of infection among all areas, the rate of decline in infection odds varied by political belief and was most pronounced in Orthodox areas, followed by those characterized by Right, Center, Left, and Arab views. Hence, among areas characterized by conservative beliefs (Right and Orthodox) less responsive to virus risk, stringent pandemic treatment controls such as economic closure were shown to be more effective.

Overall, results add to a growing body of literature suggesting that a common public signal about health risk (in our case, virus infections) or related policy treatment is differentially interpreted and acted upon depending on worldview and political belief. The estimated belief effects may derive from political or ideological imperative or bias in information processing, as discussed by [28,29,30]. Additionally, our findings are consistent with the literature showing the importance of political beliefs in the interpretation of news and response to political and economic events and policy (see, e.g., Refs. [17,31,32]). Our findings underscore the importance of targeted messaging and treatment among belief groups so as to enhance the efficacy of vaccination and related policy measures. Indeed, results show that populations less responsive to disease risk do better with more restrictive and binding pandemic crisis management. Our findings provide new evidence of the durability of political belief in the face of palpable health risks. The results underscore the importance of political belief in risk-related behaviors and the efficacy of related policy interventions. Such insights may be salient to future health policy designs and interventions in the presence of ongoing belief divergence.

## 5. Conclusions

We use comprehensive voting data from Israel to characterize small statistical areas by political belief and to assess how belief affects the response to virus risk in the determination of COVID-19 virus transmission, vaccine resistance, and treatment outcomes. All things equal, findings show that when confronted by emergent and localized virus risk, neighborhoods comprised of politically right-of-center and religious voters are associated with substantially higher odds of both vaccine resistance and virus transmission compared to their left-center counterparts. Further, the aggregate disease costs of less risk-averse behaviors are substantial. Model simulation shows the saliency of estimated belief effects: had all statistical areas responded to the virus with the more risk-averse behaviors associated with left-of-center areas, the total number of vaccinations (infections) would have increased (declined) by 15 (30) percent. Finally, results show that stringent pandemic treatment interventions such as economic closure were more effective among conservative areas that were less prone to change virus behaviors in response to prevailing COVID-19 disease risk.

Future research should assess the external validity of results as belief effects may be of first-order importance to COVID-19 variant transmission and response formulation among decision-makers globally. As only political belief data aggregated to the small statistical area geography was available for this study, we are unable to link individual political beliefs to risky health behaviors. Data permitting, it would be useful to estimate statistical models using individual data so as to corroborate the results of the statistical area analysis and shed further light on the role of worldview and political belief in the determination of pandemic-related health behaviors and outcomes. Finally, additional work is required in assessing the efficacy of nuanced, belief-targeted public health messaging in efforts to raise vaccine uptake and dampen virus spread.

## Figures and Tables

**Table 1:** Variables description and summary statistics.

Variable	Description	Mean	Std	Min	Max	Right	Center	Left	Arab	Orthodox
*Infections*	Total number of weekly infections	4.6	12.2	0	544	3.9	3.0	2.0	5.1	16.2
*Vaccinations*	Total number of weekly vaccinations	195.4	169.9	0	2334	185.9	215.7	226.0	200.6	114.6
*Ln* (*OddsInfect*)	Log odds of weekly infections	−6.17	1.89	−6.90	6.90	−6.12	−6.25	−6.42	−6.15	−5.73
*Ln* (*OddsVac*)	Log odds of weekly vaccinations	−4.79	3.72	−6.90	6.90	−4.94	−4.76	−4.55	−5.02	−4.74
*Right*	Dummy variable equals 1 for right-leaning cluster	0.38	0.48	0	1					
*Center*	Dummy variable equals 1 for center cluster	0.28	0.45	0	1					
*Left*	Dummy variable equals 1 for left-leaning cluster	0.18	0.38	0	1					
*Orthodox*	Dummy variable equals 1 for Orthodox Jewish cluster	0.10	0.30	0	1					
*Arab*	Dummy variable equals 1 for Arab cluster	0.049	0.217	0	1					
*RightCont*	Share of votes for right-leaning parties	0.37	0.18	0	0.89	0.53	0.41	0.24	0.04	0.11
*OrthodoxCont*	Share of votes for Orthodox Jewish parties	0.17	0.24	0	0.98	0.16	0.07	0.02	0.01	0.85
*ArabCont*	Share of votes for United Arab List									
*NonVoter*	Share of non-voters among those eligible to vote	0.34	0.09	0.10	0.86	0.37	0.35	0.3	0.38	0.27
*Pop*	Population size	4589	2465	1974	27,768	4279	4454	4393	5796	5917
*Density*	Population density (Pop divided by geographic area in square kilometers)	13,177	10,223	39.3	66,159	10,827	13,555	12,107	6888	26,198
*SES*	Socio-economic index score	0.22	1.01	−3.13	2.53	−0.11	0.63	1.52	−0.75	−1.59
*ProximityTA*	Distance to Tel Aviv (index)	0.68	0.93	−4.97	1.48	0.44	0.79	1.16	−0.06	0.77
*Age60*	Share of population over the age of 60	0.20	0.07	0	0.49	0.21	0.23	0.21	0.12	0.08
*Age15*	Share of population under the age of 15	0.24	0.08	0.05	0.65	0.24	0.22	0.21	0.27	0.43
*PersonHH*	Average number of persons in the household	3.18	0.83	1.50	7.10	3.13	2.94	2.74	3.82	4.66
*RoomsHH*	Average number of rooms per person	1.52	0.26	0.58	2.44	1.51	1.61	1.75	1.25	1.1

Notes: Table 1 presents summary statistics for the entire sample and sample stratified by ideological clusters.

**Table 2:** Results from the estimation of Equations (1) and (2)—log odds of vaccination and infection.

Column	(1)	(2)	(3)	(4)	(5)	(6)	(7)	(8)
Outcome Term	Vac	Vac	Vac	Infect	Infect	Infect	Infect	Infect
*Constant*	−2.523 (0.024)	−2.482 (0.333)	−2.946 (0.325)	−6.671 (0.005)	−6.394 (0.097)	−6.638 (0.071)	−6.511 (0.089)	−5.950 (0.179)
*Left*	0.520 (0.065)	−0.073 (0.091)	−0.517 (0.097)	−0.154 (0.007)	−0.065 (0.017)	0.089 (0.014)	0.50 (0.015)	−0.161 (0.071)
*Center*	0.250 (0.046)	0.014 (0.046)	−0.159 (0.054)	−0.077 (0.007)	−0.031 (0.010)	0.050 (0.008)	0.040 (0.008)	−0.016 (0.085)
*Orthodox*	−0.456 (0.075)	0.011 (0.122)	0.253 (0.128)	0.462 (0.020)	0.229 (0.028)	−0.259 (0.023)	−0.211 (0.025)	−0.318 (0.137)
*Arab*	−0.152 (0.100)	0.119 (0.108)	−0.152 (0.201)	0.001 (0.020)	−0.056 (0.022)	−0.021 (0.016)	−0.050 (0.015)	−0.775 (0.116)
*Infections_t_*_−1_			0.177 (0.018)			0.179 (0.008)	0.165 (0.009)	0.272 (0.016)
*Left* × *Infections_t_*_−1_			0.343 (0.044)			−0.168 (0.014)	−0.129 (0.014)	−0.167 (0.027)
*Center* × *Infections_t_*_−1_			0.106 (0.018)			−0.071 (0.010)	−0.072 (0.010)	−0.065 (0.030)
*Orthodox* × *Infections_t_*_−1_			−0.050 (0.017)			0.217 (0.011)	0.216 (0.014)	0.235 (0.030)
*Arab* × *Infections_t_*_−1_			0.150 (0.060)			−0.010 (0.015)	0.011 (0.021)	−0.028 (0.039)
*t*								−0.301 (0.007)
*Left* × *t*								0.149 (0.011)
*Center* × *t*								0.043 (0.011)
*Orthodox* × *t*								−0.121 (0.015)
*Arab* × *t*								0.227 (0.024)
Controls	No	Yes	Yes	No	Yes	Yes	Yes	Yes
N	25,650	25,650	25,650	51,300	51,300	51,300	27,000	6750
Number of weeks	19	19	19	39	38	38	20	5
Prob(F)	0	0	0	0	0	0	0	0
R2-overall	0.039	0.058	0.178	0.113	0.126	0.468	0.481	0.747

Notes: Columns 1–3 (4–6) present results from the estimation of vaccination (infection) Equation (1) for the period 20 December 2020–25 April 2021 (15 March–20 December 2020). Column 7 presents results from the estimation of the infection equation for the period 10 May–24 September 2020. Column 8 presents results from the estimation of infection Equation (2) for the period closure period 25 September–17 October 2020. A list of control terms and related estimation results are presented in Appendix A, Table A1. Standard errors are in parentheses.

## Data Availability

Data available upon request from paper authors.

## Appendix A

**Table A1:** Estimation results for control terms omitted from Table 2.

Column	(1)	(2)	(3)	(4)	(5)	(6)	(7)	(8)
Outcome Term	Vac	Vac	Vac	Infections	Infections	Infections	Infections	Infections
*Pop*	--	−4 × 10^−6^ (8 × 10^−6^)	−2.6 × 10^−5^ (8 × 10^−6^)	--	−5 × 10^−6^ (2 × 10^−6^)	−1.6 × 10^−5^ (3 × 10^−6^)	−1.6 × 10^−5^ (2 × 10^−6^)	−3 × 10^−5^ (6 × 10^−6^)
*Density*	--	−9 × 10^−6^ (3 × 10^−6^)	8 × 10^−6^ (3 × 10^−6^)	--	2 × 10^−6^ (1 × 10^−6^)	0.0 (0.0)	0.0 (0.0)	1 × 10^−6^ (1 × 10^−6^)
*SES*	--	0.349 (0.049)	0.407 (0.047)	--	−0.090 (0.011)	−0.055 (0.008)	−0.066 (0.009)	−0.172 (0.021)
*ProximityTA*	--	−0.015 (0.021)	−0.022 (0.020)	--	0.013 (0.004)	0.010 (0.003)	0.009 (0.004)	0.052 (0.009)
*Age*60	--	0.702 (0.460)	0.813 (0.456)	--	−0.124 (0.071)	−0.183 (0.073)	−0.238 (0.066)	0.048 (0.157)
*Age*15	--	0.928 (0.526)	0.981 (0.497)	--	−0.296 (0.121)	−0.196 (0.104)	−0.299 (0.116)	−0.064 (0.253)
*PersonHH*	--	0.049 (0.037)	0.047 (0.035)	--	0.025 (0.010)	0.016 (0.007)	0.013 (0.008)	0.035 (0.018)
*RoomsHH*	--	0.129 (0.150)	0.159 (0.146)	--	−0.007 (0.035)	0.014 (0.029)	0.021 (0.028)	0.062 (0.051)
*NonVoter*	--	−1.542 (0.305)	−1.212 (0.301)	--	−0.705 (0.133)	−0.371 (0.101)	−0.602 (0.113)	−0.867 (0.189)
Controls	No	Yes	Yes	No	Yes	Yes	Yes	Yes
N	25,650	25,650	25,650	51,300	51,300	51,300	27,000	6750
Number of weeks	19	19	19	39	38	38	20	5
Prob (F)	0	0	0	0	0	0	0	0
R2-overall	0.039	0.058	0.178	0.113	0.126	0.468	0.481	0.747

Notes: Table A1 provides estimates of X vector control variables from Equations (1) and (2) omitted from Table 2 for purposes of brevity. Note that the above controls were excluded from specifications in columns (1) and (4) of Table A1. Standard errors are in parentheses.

**Table A2:** Results from the estimation of Equations (1) and (2)—replacing belief fixed-effects with continuous belief terms.

Column	(1)	(2)	(3)	(4)	(5)	(6)	(7)	(8)
Outcome Term	Vac	Vac	Vac	Infect	Infect	Infect	Infect	Infect
*Constant*	−1.944 (0.073)	−2.320 (0.375)	−3.236 (0.370)	−6.876 (0.007)	−6.496 (0.113)	−6.517 (0.082)	−6.409 (0.100)	−6.216 (0.202)
*RightCont*	−0.608 (0.142)	−0.148 (0.188)	0.249 (0.218)	0.154 (0.016)	0.133 (0.038)	−0.023 (0.029)	0.011 (0.031)	0.769 (0.163)
*OrthodoxCont*	−1.145 0.103	−0.293 (0.305)	0.393 (0.299)	0.755 (0.021)	0.687 (0.063)	−0.232 (0.053)	−0.142 (0.053)	0.749 (0.205)
*ArabCont*	−0.826 (0.135)	−0.090 (0.236)	0.068 (0.316)	0.227 (0.023)	0.205 (0.049)	−0.056 (0.038)	−0.056 (0.037)	−0.161 (0.153)
*Infections_t_*_−1_			0.515 (0.041)			−0.051 (0.018)	−0.021 (0.018)	0.077 (0.028)
*RightCont* × *Infections_t_*_−1_			−0.422 (0.068)			0.250 (0.029)	0.177 (0.033)	0.204 (0.051)
*OrthodoxCont* × *Infections_t_*_−1_			−0.403 (0.042)			0.480 (0.020)	0.434 (0.021)	0.431 (0.045)
*ArabCont* × *Infections_t_*_−1_			−0.201 (0.081)			0.236 (0.023)	0.212 (0.028)	0.155 (0.045)
*t*								−0.082 (0.013)
*RightCont* × *t*								−0.308 (0.027)
*OrthodoxCont* × *t*								−0.377 (0.018)
*Arab* × *t*								0.013 (0.030)
Controls	No	Yes	Yes	No	Yes	Yes	Yes	Yes
N	25,650	25,650	25,650	51,300	51,300	51,300	27,000	6750
Number of weeks	19	19	19	39	38	38	20	5
Prob (F)	0	0	0	0	0	0	0	0
R2-overall	0.043	0.058	0.181	0.125	0.132	0.465	0.482	0.756

Notes: Table A2 presents results from estimations of Equations (1) and (2), replacing the belief fixed-effects with continuous belief terms, including RightCont, OrthodoxCont, and ArabCont, where those terms represent the share of votes in each statistical area for right-leaning, Orthodox, and united Arab parties, respectively. Standard errors are in parentheses.

**Table A3:** Results of estimation of Equations (1) and (2)—replacing $Infections_{t-1}$ with either $Hospitalization_{t-1}$ or $Infections_{t-2}$.

Column	(1)	(2)	(3)	(4)	(5)	(6)	(7)	(8)
Outcome Term	Vac	Infect	Infect	Infect	Vac	Infect	Infect	Infect
*Constant*	−2.506 (0.329)	−6.403 (0.090)	−6.347 (0.106)	−4.782 (0.263)	−3.047 (0.328)	−6.539 (0.081)	−6.415 (0.099)	−5.568 (0.205)
*Left*	−0.156 (0.089)	−0.047 (0.016)	−0.044 (0.015)	−0.697 (0.051)	−0.309 (0.097)	0.064 (0.014)	0.014 (0.015)	−0.277 (0.069)
*Center*	−0.014 (0.047)	−0.020 (0.008)	−0.025 (0.009)	−0.241 (0.042)	−0.060 (0.053)	0.037 (0.008)	0.019 (0.009)	−0.034 (0.072)
*Orthodox*	0.019 (0.121)	0.145 (0.027)	0.154 (0.031)	0.677 (0.085)	0.382 (0.130)	−0.177 (0.023)	−0.164 (0.027)	−0.113 (0.151)
*Arab*	0.001 (0.131)	−0.070 (0.020)	−0.110 (0.022)	−1.096 (0.097)	−0.172 (0.217)	−0.013 (0.018)	−0.060 (0.017)	−1.042 (0.135)
*Z*	0.037 (0.021)	0.129 (0.014)	0.082 (0.023)	0.124 (0.022)	0.195 (0.018)	0.117 (0.007)	0.095 (0.008)	0.184 (0.017)
*Left* × *Z*	0.599 (0.139)	−0.213 (0.031)	−0.167 (0.050)	−0.041 (0.045)	0.215 (0.047)	−0.151 (0.013)	−0.118 (0.014)	−0.161 (0.028)
*Center* × *Z*	0.098 (0.040)	−0.076 (0.029)	−0.075 (0.033)	0.002 (0.051)	0.055 (0.019)	−0.063 (0.009)	−0.066 (0.010)	−0.070 (0.029)
*Orthodox* × *Z*	0.011 (0.048)	0.308 (0.028)	0.293 (0.042)	0.029 (0.042)	−0.110 (0.017)	0.185 (0.028)	0.212 (0.015)	0.211 (0.036)
*Arab* × *Z*	0.255 (0.106)	0.010 (0.036)	0.126 (0.062)	0.006 (0.073)	0.147 (0.065)	−0.023 (0.016)	0.009 (0.021)	0.009 (0.044)
*t*				−0.375 (0.006)				−0.377 (0.007)
*Left* × *t*				0.191 (0.010)				0.192 (0.010)
*Center* × *t*				0.054 (0.010)				0.054 (0.011)
*Orthodox* × *t*				−0.113 (0.016)				−0.167 (0.015)
*Arab* × *t*				0.237 (0.026)				0.268 (0.025)
Controls	Yes	Yes	Yes	Yes	Yes	Yes	Yes	Yes
N	25,650	51,300	27,000	6,750	25,650	49,950	27,000	6750
Number of weeks	19	38	20	5	19	37	20	5
Prob (F)	0	0	0	0	0	0	0	0
R2-overall	0.070	0.173	0.199	0.702	0.147	0.324	0.352	0.720

Notes: Table A3 presents results obtained from re-estimating Equations (1) and (2), replacing $Infections_{t-1}$ with either $Hospitalization_{t-1}$ or $Infections_{t-2}$. The variable *Z* represents $Hospitalization_{t-1}$ and $Infections_{t-2}$ in columns 1–4 and 5–8, respectively. Columns 1 and 2 (5 and 6), respectively, present outcomes from the estimation of the vaccination and infection Equation (1) for the full sample; column 3 (7) presents results from the estimation of the infection Equation (1) for the period 10 May–20 September 2020, between the first and second rounds of closure; and column 4 (8) presents results from the estimation of Equation (2) for the closure period sample. For the sake of brevity, results for the control vector X are omitted and available upon request. Standard errors are in parentheses.
